# The link between atopic dermatitis and mental health outcomes across childhood: A longitudinal cohort study

**DOI:** 10.1001/jamadermatol.2021.2657

**Published:** 2021-10-01

**Authors:** Chloe Kern, Joy Wan, Kaja LeWinn, Faustine Ramirez, Yong Lee, Charles E. McCulloch, Sinéad M Langan, Katrina Abuabara

**Affiliations:** 1 Department of Dermatology, University of California San Francisco (UCSF); 2 Department of Dermatology, Perelman School of Medicine, University of Pennsylvania; 3 Department of Psychiatry and Behavioral Sciences, University of California San Francisco (UCSF); 4 Department of Pediatrics, University of California San Francisco (UCSF); 5 Department of Epidemiology and Biostatistics, University of California San Francisco (UCSF); 6 Faculty of Epidemiology and Population Health, London School of Hygiene and Tropical Medicine, London, UK

## Abstract

**Importance:**

Research has highlighted associations between atopic dermatitis (AD) and mental health conditions in adults. However, literature on the development of mental health comorbidities in children is limited despite the large burden of pediatric AD worldwide.

**Objective:**

To examine the relationship between AD and internalizing behaviors and symptoms of depression at multiple time points across childhood and adolescence, and to explore potential mediating factors including asthma/rhinitis, sleep, and inflammation.

**Design:**

Longitudinal cohort study

**Setting:**

Population-based birth cohort in the United Kingdom

**Participants:**

Children followed from birth for a mean duration of 10.0 years (SD 2.9 years) from The Avon Longitudinal Study of Parents and Children

**Exposure:**

AD annual period prevalence and disease severity assessed at 11 time points between ages 6 months and 18 years, measured by standardized questions about flexural dermatitis

**Main outcomes and measures:**

Symptoms of depression, measured using child-reported responses to the Short Moods and Feelings Questionnaire at five time points between ages 10 and 18 years; and Internalizing behaviors, measured by maternal report of the Emotional Symptoms subscore of the Strength and Difficulties Questionnaire collected at seven time points between ages 4 and 16 years.

**Results:**

Among 11,181 children, the annual period prevalence of internalizing behaviors and symptoms of depression ranged from 10.3% to 16.0% and 6.0% to 22.1%, respectively. Although mild/moderate AD was not associated with symptoms of depression, it was associated with internalizing behaviors as early as age 4 (average of 29-84% increased odds across childhood in adjusted models). Severe AD was associated with symptoms of depression (aOR 2.38, 95% CI 1.21-4.72) and internalizing symptoms (aOR 1.90, 95% CI 1.14-3.16). Sleep quality mediated some of this association, but it was not explained by differences in sleep duration, asthma/rhinitis, nor inflammatory markers (IL-6 and CRP).

**Conclusion and relevance:**

Within a population-based birth cohort in the United Kingdom, severe AD was associated with symptoms of depression and internalizing behaviors throughout childhood and adolescence. Risk of internalizing symptoms was increased even for children with mild AD beginning early in childhood, highlighting the importance of behavioral/mental health awareness in providers treating this population.

## Introduction

Atopic dermatitis (AD, also known as eczema or atopic eczema) is the most common inflammatory skin disease: globally it is estimated that AD affects up to 15%- 20% of pediatric populations and up to 10% of adult populations [1] [2]. Recent research has highlighted many important systemic comorbidities, [3] and among them, a growing body of evidence supports a strong association between AD and anxiety, depression, and suicidality [4, 5] [6]. However, most studies have been cross-sectional and focused on adults. AD often presents early in life and is common among children; therefore, additional research is needed to understand the development of mental health symptoms in younger populations to design early screening and interventions.

Pediatric depression often presents with subtle symptoms and is underdiagnosed and undertreated.[7] [8] Internalizing behaviors, (e.g. behaviors directed inward that are indicative of a child’s emotional and psychological states, including depressive behavior, anxious behavior, and somatic complaints) can be used to identify children who may require early intervention.[9, 10]

Longitudinal data with repeated assessments of AD and mental health are also needed to understand potential associations throughout childhood. The heterogeneous and waxing and waning nature of AD makes it important to examine disease course over time to account for variations in disease activity and severity. [11] Similarly, childhood and adolescence are particularly critical times for the development of mental illness and are characterized by rapid fluctuations of severity across short intervals [12].

A variety of possible mechanisms have been proposed to explain a potential association between AD and mental health outcomes that warrant additional study in children. These include associations with other atopic diseases (i.e., asthma and/or allergies), disrupted sleep, perceived social stigma, and lifestyle changes such as limited exercise because of sweat-induced itch. Multiple studies have also found that some subtypes of depression are associated with increased markers of systemic inflammation such as IL-6 and CRP [13] [14]. These biomarkers may be elevated in patients with AD [15] [16], suggesting a possible common underlying inflammatory etiology.

The objective of this study was to examine the relationship between AD and symptoms of depression at multiple time points across childhood and adolescence. Additionally, we sought to explore the role of potential mediating factors including asthma/rhinitis, sleep, and inflammation.

## Methods

We performed a longitudinal cohort study using data collected from 1990 to 2009 from the Avon Longitudinal Study of Parents and Children (ALSPAC). Informed consent for the use of data collected via questionnaires and clinics was obtained from participants following the recommendations of the ALSPAC Ethics and Law Committee at the time, and consent for biological samples has been collected in accordance with the Human Tissue Act (2004). Ethical approval for the study was obtained from the ALSPAC Ethics and Law Committee and the Local Research Ethics Committees. The present analysis was not considered human subjects research by UCSF.

### Participants

ALSPAC is an intergenerational longitudinal cohort that recruited pregnant women residing in Avon, UK with expected delivery dates between April 1^st^ 1991 and December 31^st^ 1992. They have been followed longitudinally with standardized questionnaires and clinical assessment visits. A total of 14,062 children from 14,451 pregnancies were enrolled, of which 13,988 were alive at 1 year.[17, 18] The current study sample is limited to children alive at 1 year of age with at least one completed assessment of AD and one completed mood questionnaire (n=11,181) (Figure S1). The ALSPAC website contains a fully searchable data dictionary and variable search tool: http://www.bristol.ac.uk/alspac/researchers/our-data/.

### Measures

#### Exposure

The primary exposure was a repeated measure of AD annual period prevalence assessed at 11 time points based on the following standardized question about flexural dermatitis and severity: “Has your child had an itchy, dry skin rash in the joints and creases of his/her body (eg, behind the knees, elbows, under the arms) in the past year?”, and was reported by mothers through age 14 and by the child at ages 16 and 18. To account for its chronic and relapsing course, active AD was defined as having at least 2 reports of flexural dermatitis, up to and including the time point being considered.[19] Disease severity was assessed at each time point (until age 16) by a subsequent question asking whether symptoms over the past year were “no problem, mild, quite bad, or very bad.” To ensure adequate numbers in each subcategory for analysis we combined the first two categories and termed these levels no problem/mild, moderate, and severe.

#### Outcomes

Internalizing behaviors were measured using maternal report of the Emotional Symptoms subscore of the Strength and Difficulties Questionnaire (SDQ) collected at seven time points between ages 4 and 16 years. The emotional symptoms score is based on 5 items assessing the presence of somatic symptoms (“I get a lot of headaches, stomachaches, or sickness”), cognitive symptoms (“I worry a lot”, “I am often unhappy”, “I have many fears”), and signs of low self-esteem (“I easily lose confidence”). Mothers were asked to rate their child’s behavior on each item as not true (0), somewhat true (1), or certainly true (2), and responses were totaled on a scale from 0-10 (Supplemental Methods). The SDQ has been used extensively worldwide and has been shown to have good internal consistency and retest stability.[20] In accordance with other studies, a score > 90^th^ percentile of the sample was used as a binary indicator of internalizing behavior, corresponding to an emotional symptoms score ≥ 4.[20]

Depression was measured using child-reported responses to the Short Moods and Feelings Questionnaire (SMFQ) at five time points between ages 10 and 18 years. The SMFQ has been validated for use in identifying signs and symptoms of depression in children and adolescents aged 6 to 19 [21] and has been shown to have good internal validity for examining depressive symptoms over time within ALSPAC as measured by Cronbach’s alpha (mean Cronbach α: 0.86) [22]. SMFQ was analyzed as a binary outcome with a score of 11 or higher used to identify individuals with symptoms of depression, in accordance with other studies in the literature [22] [23].

Associations with clinician-diagnosed depression and anxiety based on the Development and Well-Being Assessment (DAWBA), available only at age 7, were also assessed.

#### Covariates

Potential confounding factors were identified from a review of the literature and a directed acyclic graph was constructed to highlight assumptions about relationships between variables in the models (Figure S2).

Potential confounding variables included: (1) sociodemographic characteristics: child sex, age, and ethnicity, and maternal age at delivery; (2) measures of socioeconomic status and (3) maternal pre- and post-natal depression.

Potential mediating variables included: time-updated comorbid atopy (parent-reported asthma or allergic rhinitis symptoms), sleep disturbance (parent-reported sleep duration and sleep quality), and inflammatory biomarkers (serum IL-6 and serum CRP), as described in detail in the Supplemental Methods. These were selected based on prior literature and availability in the ASLPAC cohort.

#### Statistical Analysis

We performed cross-sectional analyses using logistic and linear regression models to examine the association between AD symptoms over the past year and each of the binary mental health outcomes at available ages. In pre-planned sensitivity analyses we also examined the outcomes as continuous numerical scores. After confirming consistency of the cross-sectional results across ages, we performed longitudinal analyses to determine the average subject-specific effects across time points using mixed-effects models with random intercepts for each individual. Models were adjusted for the minimally sufficient adjustment set of time-varying covariates as determined by the directed acyclic graph as described above. All models were repeated with AD modeled as a categorical rather than binary variable to determine if the strength of association varied by disease severity.

We assessed whether potential mediators (asthma, allergic rhinitis, sleep duration and quality, and inflammatory biomarkers) were associated with AD and depression. For variables that were associated with both (p<0.05), we examined the indirect effect and calculated the proportion of the total effect accounted for by the mediator using the stata medeff package that builds on the Baron and Kenny approach by allowing for binary outcomes [24, 25]. Finally, to test for effect modification between AD and asthma, allergic rhinitis, and sex, we individually tested each covariate by including an interaction term with AD.

#### Missing Data

As has been previously described, there was both intermittent missing data and attrition from the cohort over time [17], therefore the amount of missing data varied by age. Mixed models were used to accommodate missingness in outcome data (SMFQ, SDQ), and multivariable analyses were restricted to individuals with complete covariate data. Covariate data like markers of social class were most likely to be missing at random (as missingness depends on the actual values), and complete case analysis is valid where missingness is independent of the outcome, conditional on the model covariates.[26]

Statistical analyses were performed using Stata, version 14.2 (StataCorp Inc).

## Results

The study sample was composed of 11,181 individuals (51.2% male) with at least one response to a question about AD and at least one response to a question about symptoms of depression (8,458 individuals for the SMFQ analysis, 10,785 individuals for the SDQ analysis). Children were followed from birth for a mean duration of 10.0 years (standard deviation 2.9 years). From age 3 to 18 years, the annual period prevalence of active AD decreased from 19.1% to 14.5%, and the proportion reporting moderate or severe symptoms over the prior 12 months ranged from 21.8-40.1% (Table S1). The amount of missing covariate data ranged from 0.1-20.6% as shown in Table 1, and the amount of missing outcome data increased over time due to attrition as shown in Tables 2 and S4.

### Symptoms of depression as measured by the self-reported SMFQ

The period prevalence of symptoms of depression as measured by the SMFQ increased from 6% at age 10 years to 21.6% at age 18 years. Children with symptoms of depression were more likely to be female, from a higher social class, have a parent with a higher level of education, and be from families reporting fewer financial difficulties (Table 1). In adjusted longitudinal mixed models that incorporated all timepoints in childhood and adolescence, the odds of depressive symptoms for children with AD compared to those without AD was 1.14, 95% CI 0.93-1.40 (Table 3). Increasing severity of AD was consistently associated with increasing odds of symptoms of depression (Figure 1) and followed a linear trend (p-value 0.029). The subject-specific odds of symptoms of depression for severe AD across all time points was 2.38, 95% CI 1.21-4.72 (Table 3). In a sensitivity analysis using numerical SMFQ scores we found that participants with AD had a 0.3 point higher SMFQ score with any AD (95% CI 0.08-0.55), and a 2 point higher SMFQ score with severe AD (95% CI 1.23-2.78, Table S3).

### Internalizing behaviors as measured by the emotional symptom score of the Parent-reported SDQ

Period prevalence of internalizing behavior, as measured by a score of 4 or above on the emotional symptom’s subscale of the SDQ, ranged from 10.3% to 16.0% between ages 4 and 16 years, and we did not observe a trend with age (Table S5). In cross-sectional analyses, children with AD consistently had higher odds of internalizing behavior, and the subject-specific effect averaged across all time points showed a 42% increase in the odds of internalizing symptoms among those with active AD (aOR 1.41, 95% CI 1.17-1.70, Table 3). We also observed a dose-response effect according to AD severity levels (Table 3). Using the numerical ‘emotional symptoms’ score, we found similar results.

We did not find evidence of interactions between AD and asthma, allergic rhinitis, and child’s sex in any of the cross-sectional associations for either outcome.

To assess the impact of missing covariate data, we compared the main mixed model results adjusted for all covariates to a model that adjusts only for covariates with <1% missingness and found similar results for both SMFQ and SDQ (Table S6).

### Clinician-diagnosed depression and anxiety

A total of 1,356 children in the cohort at age 7 years had active AD, and N=273, 3.7% received a clinician diagnosis of any anxiety or depression disorder based on the DAWBA, which represents a 51% increased odds (adjusted OR 1.51, 95% CI 0.97-2.36, Table S8).

### Associations with potential mediators

#### Biomarkers of inflammation

In cross-sectional analyses, we did not find associations between IL-6 and AD or symptoms of depression (as measured by the SMFQ) at age nine years, nor with CRP at ages 9, 14 and 16 years. (Table S8).

#### Atopy

We did not find associations between asthma or allergic rhinitis and both AD and symptoms of depression at available timepoints.

#### Sleep

We did not find associations between sleep duration and internalizing behavior at any of the available timepoints (ages 4, 7, 9, 11, and 14 years). However, associations between sleep quality and both AD and internalizing behavior were seen at all available timepoints (ages 4, 7, and 9 years) (Tables S9). Sleep quality mediated 11.3% (95% CI 7.0 – 23.1) of the association between AD and internalizing behavior at age 4 years, and 14.7% (95%CI 8.6-40.2) at 7 years (Table S10). The analyses at age 9 and by severity were limited by wide confidence intervals.

## Discussion

Using data from nearly two decades of follow-up from a population-based birth cohort, we found that severe AD was associated with an approximately two-fold increased odds of symptoms of depression and internalizing behavior. Mild and moderate AD were associated with a 30-85% increased risk of internalizing behavior at ages 4-16, but not with an increased risk of symptoms of depression at ages 9-18. These findings are important because childhood depression and internalizing behavior have been linked to adult depression and anxiety, other psychiatric disorders, and poor general health. [27] [28] [29] [30] [31]

Our study adds to limited literature on AD and mental health in childhood, which has been primarily cross-sectional. Our results showing a dose-response relationship with more severe disease are similar to other studies [32] [33] [34] [5]. Two longitudinal birth cohorts examined AD disease trajectories and mental health at age 10, [35] [36] ours is unique in its repeated assessments of both AD and mental health outcomes at multiple ages.

Our results suggest that awareness of mental health among children with AD is important across childhood. Associations between AD and internalizing behavior were present at 4 years, the youngest age assessed. Consistent with prior studies, the prevalence of internalizing behaviors remained fairly stable from age 4 to 16 years,[37] [38] and the strength of association with AD remained similar across ages, suggesting that children are at increased risk at all time points, including in early childhood. Early behavioral/mental health assessment and intervention may have multiplicative effects as mental health comorbidity has been shown to exacerbate AD flares and limit consistent adherence to treatment plans.[39] [40] Additionally, adolescence has been identified as a particularly vulnerable time for rapid development and severity of depressive symptoms, and previous studies have reported that adolescents with AD and pruritus were found to have three times the odds of suicidal ideation.[22] [41]

Several potential mechanisms for an association between AD and depression have been proposed. We were able to examine whether some of these factors helped to explain the observed associations via mediation analyses. Patients with eczema often have comorbid asthma and rhinitis, but we did not find evidence that the association with depression was explained by these conditions. We also investigated sleep disturbances because we have previously found poor sleep quality among children with AD, and because poor sleep has been shown to contribute to higher rates of mental health disorders.[42] [43] [4] In this cohort, we found that impaired sleep quality explains a small proportion of the relationship between AD and internalizing symptoms (at 4, 7, and 9 years), though confidence intervals were wide due to relatively small sample sizes of patients with severe disease. Mediation by sleep quality warrants additional study in other populations.

Limitations of our study warrant consideration. Internalizing behaviors, symptoms of depression, and AD were all based on parental or self-report. We used measures based both on maternal report (SDQ) and child self-report (SMFQ) and controlled for maternal pre- and/or post-natal depression and anxiety which may bias maternal report. Others have found that children and adolescents report more internalizing symptoms with higher predictive value compared to their parents, [44] [45] [46] so the use of parent reported SDQ might lead to an underestimation of the prevalence of internalizing behavior. Reassuringly, we found similar overall results using clinician diagnoses based on the DAWBA, but this measure was only available at age 7, and our results had wide confidence intervals due to the low rate of clinician-diagnosed outcomes at this age. Studies have also found that parental/self-report of AD approximate physician-diagnosis and assessment.[47] Another limitation relates to ethnic diversity. Though prior work has shown that the ALSPAC cohort is representative of the UK population, [17] additional research is needed in diverse settings.

In conclusion, this longitudinal analysis offers evidence of an association between AD and symptoms of depression and internalizing behavior beginning in early childhood. As many new AD therapies are being brought to market, it is important to study the impact of new therapies on sleep and mental health outcomes among pediatric patients.[48] The large and increasing burden of pediatric mental illness[49] highlights the importance of clinician awareness of the psychosocial needs of children and adolescents with AD.

## Supplementary Material

Supplementary

## Figures and Tables

**Figure:**
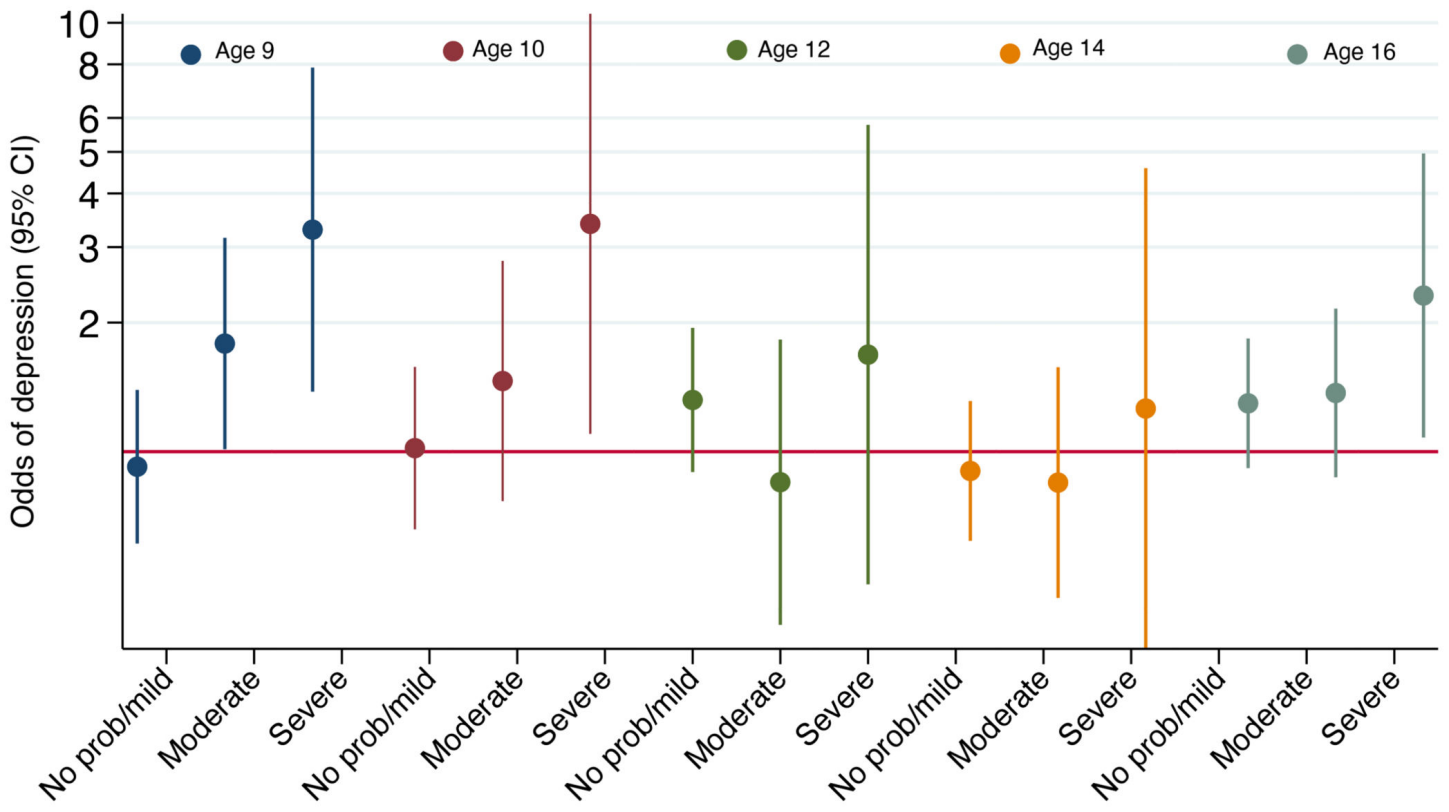
Reference group: ‘Never’ Atopic Dermatitis

**Table 1:** Cohort Characteristics

Variable	No. (% of Total)	Total (% missing^1^)	Subgroups by outcome (Depressive Mood Disorder Symptoms)	
			With^2^ (n=4,710)	Without (n=6,471)
Male sex	5,721 (51.2)	11,168 (0.1)	2,032 (43.1)	3,696 (57.1)
White ethnicity	9,935 (95.7)	10,386 (7.1)	4,251 (90.3)	5,684 (87.8)
Atopic dermatitis ever	4,456(40.6)	10,967 (1.9)	2,167 (46.0)	2,289 (35.4)
Asthma ever	3,199 (28.9)	11,074 (1.0)	1,630 (34.6)	1,569 (24.2)
Allergic rhinitis ever	1,372 (13.6)	10,067 (10.0)	821 (17.4)	551 (8.5)
Maternal age at delivery, No. (%), y				
≤ 20	585 (5.2)	11,181 (0)	225 (4.7)	360 (3.9)
21-34	9,386 (83.9)		3,944 (83.7)	5,442 (84.1)
≥ 35	1,210 (10.8)		541 (11.5)	669 (10.3)
Maternal pre- & post-natal, No. (%):				
Depression	2,733 (24.7)	11,087 (0.8)	1,433 (30.4)	1,300 (20.1)
Anxiety	3,740 (33.8)	11,080 (0.9)	1,948 (41.4)	1,792 (27.7)
Combined parent highest educational level^3^,				
No. (%)		10,613 (5.1)		
CSE/none	1,183 (11.1)		460 (9.8)	723 (11.2)
Vocational	675 (6.4)		282 (6.0)	393 (6.1)
O level	2,829 (26.7)		1,192 (25.3)	1,637 (25.3)
A level	3,500 (33.0)		1,469 (31.2)	2,031 (31.4)
Degree	2,426 (22.9)		1,127 (23.9)	1,299 (20.1)
Maternal social class, No. (%)		8,874 (20.6)		
Unskilled	171 (1.9)		75 (1.6)	96 (1.5)
Partly skilled	823 (9.9)		355 (7.5)	468 (7.2)
Skilled manual	651 (9.3)		258 (5.5)	393 (6.1)
Skilled nonmanual	3,789 (42.7)		1,606 (34.1)	2,183 (33.7)
Managerial and technical	2,876 (32.4)		1,291 (27.4)	1,585 (24.5)
Professional	560 (6.3)		243 (5.2)	317 (4.9)
Financial Difficulties quartile, No. (%)		10,320 (7.7)		
Lowest	3,874 (37.5)		1,537 (32.6)	2,337 (36.1)
Mild	2,582 (25.0)		1,093 (23.2)	1,489 (23.0)
Moderate	1,952 (18.9)		859 (18.2)	1,093 (16.9)
Highest	1,912 (18.5)		923 (20.0)	989 (15.3)
Household crowding index^4^, No. (%)		10,594 (5.2)		
<0.5	4,701 (44.4)		2,055 (43.6)	2,646 (40.9)
>0.5-0.75	3,353 (31.6)		1,387 (29.4)	1,966 (30.4)
>0.75-1	1,973 (18.6)		825 (17.5)	1,148 (17.7)
>1	567 (5.4)		225 (4.8)	342 (5.3)

1 Percentages refer to the proportion missing from 11,181 - all subjects who had at least one measurement of ‘Symptoms of Depression’ and/or ‘Internalizing Behavior’ and/or diagnosis of ‘Any Depression Disorder’ and/or ‘Any Anxiety Disorder’

2 Defined as positive response at any time point to any of the following: ‘Symptoms of Depression’ and/or ‘Internalizing Behavior’ and/or positive diagnosis of ‘Any Depression Disorder’ and/or positive diagnosis of ‘Any Anxiety Disorder’ ever from age 3y to 18y

3 Educational level: Certificate of secondary education (CSE); Ordinary-level (O-level) is a higher subject-specific qualification generally obtained at age 16 years; Advanced level (A-level) is a subject-specific qualification generally obtained at age 18 years and required for university entry.

4 Household crowding index is defined as the number of people in the household per room (excluding bathrooms and toilets)

**Table 2:** Self-reported Short Moods and Feelings Questionnaire (SMFQ) Distribution

Age (y)	Sample Size	mean (SD)	median	IQR	Missing, No. (%)	Total number above SMFQ threshold (≥11)	% with AD above the SMFQ threshold	% without AD above the SMFQ threshold
10	6,986	4.04 (3.53)	3	5	1,472 (17.4)	420	5.3	4.4
12	6,371	3.97 (3.84)	3	4	2,087 (24.7)	446	6.1	4.7
14	5,721	4.91 (4.47)	4	5	2,737 (32.4)	663	9.0	8.1
16	4,780	5.89 (5.62)	4	6	3,678 (43.5)	857	24.2	16.3
18	3,191	6.79 (5.90)	5	8	5,267 (62.3)	688	25.4	19.2

The 13-item Short Moods and Feelings Questionnaire score ranges from 0 to 26. A score of 11 or higher on the self-reported SMFQ is used as an indicator for diagnosis of depression in accordance with other studies

**Table 3:** Emotional Symptoms Score (from the parent-reported Strength and Difficulties Questionnaire) Distribution

Age (y)	Sample Size	mean	SD	median	IQR	Number above SDQ threshold^1^	% above SDQ threshold^1^	% with AD above the SDQ threshold	%without AD above the SDQ threshold	Missing, No. (%)
4	9,383	1.45	1.51	1	2	974	10.3%	12.2%	8.5%	1,402 (13.0)
7	8,196	1.49	1.66	1	2	1,031	12.6%	14.8%	10.7%	2,589 (24.0)
9	7,533	1.67	1.82	1	3	1,202	16.0%	15.4%	12.6%	3,252 (30.2)
11	7,385	1.47	1.74	1	2	952	12.9%	12.7%	9.7%	3,400 (31.5)
12	6,743	1.43	1.71	1	2	796	11.8%	13.9%	10.0%	4,402 (40.8)
14	6,740	1.43	1.72	1	2	821	12.2%	13.0%	9.5%	4,045 (37.5)
16	5,433	1.49	1.85	1	2	743	13.7%	14.5%	8.5%	5,352 (50.0)

1 defined as an emotional symptoms score of ≥4 on the Strength and Difficulties Questionnaire

**Table 4:** Longitudinal Associations with Self-reported Short Moods and Feelings Questionnaire (SMFQ) and with Parent-reported Strength and Difficulties Questionnaire (SDQ)

Mental health outcome (questionnaire)	Disease Activity & Severity		Odds Ratio (95% CI)			
			Unadjusted		Adjusted			
**Symptoms of Depression^1^** (SMFQ)	Never Any definite AD		1 [Reference] 1.35 (1.13-1.61)		1 [Reference] 1.14 (0.93-1.40)	
		No problem/mild Moderate Severe		1.24 (1.00-1.53) 1.48 (1.09-2.02) 2.51 (1.44-4.35)		1.13 (0.88-1.46) 1.13 (0.78-1.65) 2.38 (1.21-4.72)
**Internalizing Behavior^2^** (SDQ)	Never Any definite AD		1 [Reference] 1.60 (1.39-1.83)		1 [Reference] 1.41 (1.17-1.70)	
		No problem/mild Moderate Severe		1.52 (1.31-1.76) 1.81 (1.48-2.22) 1.94 (1.35-2.79)		1.29 (1.06-1.57) 1.84 (1.40-2.41) 1.90 (1.14-3.16)

1 Subject-specific average effect from adjusted multivariate mixed effects regression models including 8,458 individuals and 6 timepoints (9, 10, 12, 14, 16, and 18 years of age). All models were adjusted for child sex, age, and ethnicity, maternal age at delivery, educational qualification (highest of either parent), social class based on occupation (highest of either parent), household crowding index, financial difficulties score, maternal pre- or post-natal depression, maternal pre- or post-natal anxiety and comorbid atopic diseases (asthma and allergic rhinitis)

2 Subject-specific average effect from adjusted multivariate mixed effects regression models including 10,785 individuals and 7 timepoints (4, 7, 9, 11, 12, 14, and 16 years of age).

## Key Points

### Question

Is atopic dermatitis (AD) associated with symptoms of depression and internalizing behaviors throughout childhood and adolescence?

### Findings

Severe AD was associated with about a two-fold increase in the likelihood of symptoms of depression and internalizing symptoms across childhood. Although mild/moderate AD was not associated with symptoms of depression, it was associated with internalizing behaviors as early as age 4.

### Meaning

Because AD is common and associated with mental health comorbidities beginning in early childhood, providers should consider early screening, and future research should target mechanisms and treatments.

## Abbreviations

AD: Atopic Dermatitis
ALSPAC: Avon Longitudinal Study of Parents and Children
SMFQ: Short Moods and Feelings Questionnaire
SDQ: Strength and Difficulties Questionnaire
