# Supplementary material for: The link between atopic dermatitis and mental health outcomes across childhood: A longitudinal cohort study
Source: JAMA Dermatol. Author manuscript; Available in PMC 2021 Oct 22. (PMC8411354; doi:10.1001/jamadermatol.2021.2657)
Supplement: Supplementary [file EMS134124-supplement-Supplementary.docx]

**Supplementary Online Content**

Kern C, Wan J, LeWinn K, et al. Association of atopic dermatitis and mental health outcomes across childhood: a longitudinal cohort study. *JAMA Dermatol*. Published online July 21, 2021. doi:10.1001/jamadermatol.2021.2657

**eMethods.** Definition of outcomes and mediating variables

**eTable 1.** Atopic Dermatitis Distribution

**eTable 2.** Cross-sectional Associations with Internalizing Behaviors (SDQ)

**eTable 3.** Cross-sectional Associations with Symptoms of Depression (SMFQ)

**eTable 4.** Cross-sectional Associations with Symptoms of Depression (SMFQ) Modeled as a Numerical Score

**eTable 5.** Longitudinal Associations with Symptoms of Depression (SMFQ) and Internalizing Behaviors (SDQ) by Missing Data Status

**eTable 6.** Cross-sectional Associations with Clinician Diagnoses of Depression and Anxiety (DAWBA)

**eTable 7.** Associations Between Inflammatory Biomarkers and Symptoms of Depression (SMFQ) and Atopic Dermatitis

**eTable 8.** Associations Between Sleep Quality and Internalizing Behaviors (SDQ) and Atopic Dermatitis

**eTable 9.** Mediation by Sleep Quality of Associations Between Atopic Dermatitis and Internalizing Behaviors (SDQ)

**eFigure 1.** Study Participant Flow Diagram

**eFigure 2.** Directed Acyclic Graph

**eReferences**

This supplementary material has been provided by the authors to give readers additional information about their work.

**eMethods**

Symptoms of depression were measured using child-reported responses to the Short Moods and Feelings Questionnaire (SMFQ) at ages 10, 12, 14, 16, and 18 years. The SMFQ consists of 13 phrases relating to the feelings and actions of participants in the previous two weeks, which they are asked to rate as ‘most of the time’, ‘some of the time’ or ‘not at all’ (scoring 2, 1, or 0, respectively). SMFQ total scores are calculated by summing across each of the 13 items to create a score ranging between 0 and 26 for each participant, with a higher score on the SMFQ corresponding to a greater level of depressive symptoms. Questionnaires were submitted via mail and were returned at various points over the year. Because atopic dermatitis (AD) was measured at ages 9 and 11, but not age 10, cross-sectional analyses were repeated using AD data from age 9 with SMFQ data from age 10 and AD data from age 11 with SMFQ data from age 10.

Internalizing behaviors were measured using maternal report of the Emotional Symptoms subscale of the Strength and Difficulties Questionnaire (SDQ) collected at ages 4, 7, 9, 11, 12, 14, and 16 years. The emotional symptoms score is based on 5 items assessing the presence of somatic symptoms (“I get a lot of headaches, stomachaches, or sickness”), cognitive symptoms (“I worry a lot”, “I am often unhappy”, “I have many fears”), and signs of low self-esteem (“I easily lose confidence”). Mothers were asked to rate their child’s behavior on each item as not true (0), somewhat true (1), or certainly true (2), and responses were totaled on a scale from 0-10. In a large UK study, children with 4/5 mother-reported emotional symptoms had an increased risk for a DSMIV diagnosis of depression or anxiety (OR 11.7, 95% CI: 9.5-14.2) [1].

Potential mediating variables included: time-updated comorbid atopy (parent-reported asthma or allergic rhinitis symptoms at ages 4, 7, 9, 10, 11, 12, 14, 16 and 18 years), sleep disturbance (sleep duration based on maternal report of usual bedtime and wake time at ages 4, 7, 10, 12, and 16 years, and sleep quality based on a composite score of 4 maternal-reported sleep quality measures at ages 4, 7, and 9 years), and the two inflammatory biomarkers with available data: serum IL-6 (at age 9 only) and serum CRP (at ages 9, 14, and 16). Of note, inflammatory markers were collected at a clinic visit which was not necessarily on the same day as the questionnaires with the SDQ and SMFQ, which were collected via mail. Nighttime sleep duration was calculated on the basis of maternal report of the time the child usually went to sleep and usually woke up in the morning.^2^ Sleep quality was measured using 4 standardized questions asking about regular nighttime awakenings (≥1 per night), regular early morning awakenings, difficulty falling asleep, and nightmares over the past year. Responses were combined into a composite sleep-quality score ranging from 0 to 4. Coefficients represent the predicted unit increase in composite sleep-quality score (ranging from 0-4) among those with atopic dermatitis.

**eTable 1.** Atopic Dermatitis Period Prevalence and Severity Distribution

| Time point (years) | Total respondents | Never reported flexural dermatitis | | Inactive AD^1^ | | Maybe AD^2^ | | Active AD,  No problem | | Active AD,  Mild | | Active AD, Moderate | | Active AD, Severe | |
| --- | --- | --- | --- | --- | --- | --- | --- | --- | --- | --- | --- | --- | --- | --- | --- |
|  |  | **Number** | **%** | **Number** | **%** | **Number** | **%** | **Number** | **%** | **Number** | **%** | **Number** | **%** | **Number** | **%** |
| 3 | 9, 634 | 4,454 | 46.2 | 595 | 6.2 | 2,673 | 27.8 | 177 | 1.8 | 1,000 | 10.4 | **573** | **6.0** | **162** | 1.7 |
| 4 | 9,619 | 4,179 | 43.5 | 1,122 | 11.7 | 2,457 | 25.5 | 179 | 1.9 | 1,041 | 10.8 | 497 | 5.2 | 144 | 1.5 |
| 5 | 9,134 | 3,724 | 40.8 | 1,273 | 13.9 | 2,209 | 24.2 | 187 | 2.1 | 1,153 | 12.6 | 475 | 5.2 | 113 | 1.2 |
| 6 | 8,425 | 3,267 | 38.8 | 1,567 | 18.6 | 1,934 | 23.0 | 176 | 2.1 | 1,028 | 12.2 | 369 | 4.4 | 84 | 1.0 |
| 7 | 8,407 | 3,200 | 38.1 | 1,834 | 21.8 | 1,853 | 22.0 | 160 | 1.9 | 968 | 11.5 | 311 | 3.7 | 81 | 1.0 |
| 9 | 7,857 | 2,850 | 36.3 | 1,818 | 23.1 | 1,720 | 23.0 | 173 | 2.2 | 926 | 11.8 | 298 | 3.8 | 72 | 0.9 |
| 11 | 7,398 | 2,592 | 35.0 | 2,023 | 27.4 | 1,571 | 21.2 | 107 | 1.4 | 830 | 11.2 | 242 | 3.3 | 33 | 0.5 |
| 12 | 7,037 | 2,354 | 33.4 | 1,940 | 27.6 | 1,465 | 20.8 | 137 | 2.0 | 862 | 12.2 | 229 | 3.3 | 50 | 0.7 |
| 14 | 6,693 | 2,217 | 33.1 | 2,218 | 33.1 | 1,402 | 21.0 | 63 | 0.9 | 567 | 8.5 | 189 | 2.8 | 37 | 0.6 |
| 16 | 4,813 | 1,451 | 30.2 | 1,637 | 34.0 | 1,032 | 21.4 | 61 | 1.3 | 354 | 7.4 | 213 | 4.4 | 65 | 1.4 |
| 18 | 3,181 | 941 | 29.6 | 1,158 | 36.4 | 621 | 16.5 | 461 (14.5)^3^ | | | | | | | |

**Notes:** AD was defined as at least two reports of flexural dermatitis. ^1^ ‘Inactive AD’ refers to at least one prior report of flexural dermatitis, but no current report at that age. ^2^ ‘Maybe AD’ refers to the first report of flexural dermatitis. ^3^ No atopic dermatitis severity data were available for age 18 years.

**e Table 2.** Cross-sectional Associations with Internalizing Behaviors (SDQ)

| **Age, y** | **Symptoms of internalizing behavior, No. (%)** | **AD activity and severity** | **Odds Ratio (95% CI)** | |
| --- | --- | --- | --- | --- |
|  |  |  | **Unadjusted** | **Adjusted^1^** |
| 4  7  9  11  12  14  16 | 974 (10.3)  1031 (12.6)  1202 (16.0)  952 (12.9)  796 (11.8)  821 (12.2)  743 (13.7) | Never  Any definite AD  No problem/mild  Moderate  Severe  Never  Any definite AD  No problem/mild  Moderate  Severe  Never  Any definite AD  No problem/mild  Moderate  Severe  Never  Any definite AD  No problem/mild  Moderate  Severe  Never  Any definite AD  No problem/mild  Moderate  Severe  Never  Any definite AD  No problem/mild  Moderate  Severe  Never  Any definite AD  No problem/mild  Moderate  Severe | 1 [Reference]  1.46 (1.22-1.75)  1.38 (1.12-1.70)  1.56 (1.17-2.08)  1.91 (1.18-3.09)  1 [Reference]  1.44 (1.21-1.73)  1.32 (1.07-1.61)  1.78 (1.30-2.44)  2.11 (1.20-3.70)  1 [Reference]  1.22 (1.01-1.46)  1.07 (0.87-1.31)  1.80 (1.32-2.45)  1.43 (0.77-2.65)  1 [Reference]  1.31 (1.06-1.63)  1.32 (1.05-1.67)  1.11 (0.72-1.70)  2.63 (1.10-6.24)  1 [Reference]  1.48 (1.20-1.83)  1.35 (1.07-1.70)  1.96 (1.35-2.83)  2.30 (1.13-4.69)  1 [Reference]  1.34 (1.05-1.72)  1.32 (1.00-1.73)  1.30 (0.82-2.06)  1.86 (0.76-4.57)  1 [Reference]  1.86 (1.40-2.48)  1.70 (1.20-2.39)  2.08 (1.36-3.19)  2.40 (1.16-4.97) | 1 [Reference]  1.49 (1.21-1.82)  1.41 (1.11-1.78)  1.58 (1.14-2.18)  1.97 (1.15-3.38)  1 [Reference]  1.34 (1.07-1.65)  1.20 (0.95-1.53)  1.71 (1.19-2.46)  1.90 (0.97-3.72)  1 [Reference]  1.06 (0.85-1.32)  0.92 (0.72-1.17)  1.63 (1.14-2.33)  1.30 (0.63-2.70)  1 [Reference]  1.27 (0.97-1.65)  1.29 (0.98-1.71)  1.05 (0.62-1.76)  1.99 (0.69-5.74)  1 [Reference]  1.33 (1.04-1.69)  1.19 (0.91-1.55)  1.82 (1.19-2.77)  2.52 (1.10-5.78)  1 [Reference]  1.14 (0.84-1.53)  1.04 (0.75-1.46)  1.31 (0.77-2.24)  1.80 (0.69-4.69)  1 [Reference]  1.58 (1.12-2.22)  1.54 (1.04-2.30)  1.54 (0.93-2.54)  2.37 (1.00-5.61) |

Notes: ^1^ Models were adjusted for child sex, and ethnicity, maternal age at delivery, educational qualification (highest of either parent), social class based on occupation (highest of either parent), household crowding index, financial difficulties score, maternal pre- or post-natal depression, maternal pre- or post-natal anxiety and comorbid atopic diseases (asthma and allergic rhinitis).

**eTable 3.** Cross-sectional Associations with Symptoms of Depression (SMFQ)

| **Age, y** | **Symptoms of depression, No. (%)** | **AD activity and severity** | **Odds Ratio (95% CI)** | |
| --- | --- | --- | --- | --- |
|  |  |  | **Unadjusted** | **Adjusted^1^** |
| 9  10  12  14  16  18^2^ | 420 (6.0)  420 (6.0)  446 (7.0)  663 (11.6)  857 (17.9)  688 (21. 6) | Never  Any definite AD  No problem/mild  Moderate  Severe  Never  Any definite AD  No problem/mild  Moderate  Severe  Never  Any definite AD  No problem/mild  Moderate  Severe  Never  Any definite AD  No problem/mild  Moderate  Severe  Never  Any definite AD  No problem/mild  Moderate  Severe  Never  Any definite AD | 1 [Reference]  1.22 (0.91-1.66)  0.99 (0.70-1.41)  1.63 (0.98-2.71)  3.53 (1.74-7.14)  1 [Reference]  1.14 (0.82-1.58)  1.00 (0.68-1.45)  1.36 (0.76-2.43)  3.20 (1.09-9.42)  1 [Reference]  1.28 (0.94-1.75)  1.32 (0.94-1.84)  1.15 (0.62-2.14)  1.70 (0.59-4.85)  1 [Reference]  1.03 (0.77-1.37)  1.02 (0.74-1.42)  1.04 (0.61-1.76)  1.15 (0.34-3.90)  1 [Reference]  1.64 (1.31-2.05)  1.43 (1.09-1.88)  1.80 (1.28-2.53)  2.50 (1.45-4.30)  1 [Reference]  1.41 (1.08-1.83) | 1 [Reference]  1.17 (0.82-1.67)  0.92 (0.61-1.39)  1.79 (1.01-3.15)  3.29 (1.38-7.86)  1 [Reference]  1.17 (0.79-1.72)  1.02 (0.66-1.58)  1.46 (0.78-2.79)  3.40 (1.01-10.50)  1 [Reference]  1.23 (0.85-1.78)  1.32 (0.90-1.94)  0.85 (0.39-1.83)  1.68 (0.49-5.78)  1 [Reference]  0.90 (0.64-1.26)  0.90 (0.62-1.31)  0.85 (0.46-1.57)  1.26 (0.35-4.58)  1 [Reference]  1.40 (1.04-1.88)  1.30 (0.92-1.84)  1.37 (0.87-2.16)  2.31 (1.08-4.96)  1 [Reference]  1.24 (0.71-2.15) |

**Notes: ^1^**Models were adjusted for child sex, and ethnicity, maternal age at delivery, educational qualification (highest of either parent), social class based on occupation (highest of either parent), household crowding index, financial difficulties score, maternal pre- or post-natal depression, maternal pre- or post-natal anxiety and comorbid atopic diseases (asthma and allergic rhinitis). ^2^ No AD severity data were available at age 18 years.

**eTable 4.** Cross-sectional Associations with Symptoms of Depression (SMFQ) Modeled as a Numerical Score

| **Age, y** | **Symptoms of depression, No. (%)** | **AD activity and severity** | **Coefficient (95% CI)** | |
| --- | --- | --- | --- | --- |
|  |  |  | **Unadjusted** | **Adjusted^2^** |
| 9  10  12  14  16  18^3^ | 420 (6.0)  420 (6.0)  446 (7.0)  663 (11.6)  857 (17.9)  688 (21. 6) | Never  Any definite AD  No problem/mild  Moderate  Severe  Never  Any definite AD)  No problem/mild  Moderate  Severe  Never  Any definite AD  No problem/mild  Moderate  Severe  Never  Any definite AD  No problem/mild  Moderate  Severe  Never  Any definite AD  No problem/mild  Moderate  Severe  Never  Any definite AD | 0 [Reference]  0.28 (0.03-0.53)  0.14 (-0.14-0.41)  0.66 (0.18-1.14)  1.10 (0.21-2.00)  0 [Reference]  0.10 (-0.16-0.35)  -0.06 (-0.34-0.22)  0.50 (0.01-0.99)  1.33 (0.04-2.63)  0 [Reference]  0.49 (0.19-0.79)  0.49 (0.17-0.82)  0.38 (-0.20-0.96)  1.30 (0.15-2.46)  0 [Reference]  0.29 (-0.11-0.69)  0.29 (-0.15-0.74)  0.35 (-0.37-1.08)  0.19 (-1.55-1.94)  0 [Reference]  1.77 (1.26-2.28)  1.31 (0.70-1.92)  2.14 (1.32-2.96)  3.33 (1.92-4.74)  0 [Reference]  1.37 (0.71-2.03) | 0 [Reference]  0.21 (-0.07-0.49)  0.08 (-0.22-0.38)  0.62 (0.09-1.15)  0.98 (-0.06-2.01)  0 [Reference]  0.12 (-0.17-0.41)  -0.03 (-0.34-0.29)  0.54 (-0.01-1.08)  1.61 (0.20-3.02)  0 [Reference]  0.41 (0.08-0.75)  0.42 (0.06-0.78)  0.21 (-0.438-0.85)  1.67 (0.33-3.00)  0 [Reference]  0 (-0.45-0.43)  -0.07 (-0.56-0.43)  0.07 (-0.72-0.86)  0.87 (-1.03-2.77)  0 [Reference]  1.05 (0.45-1.64)  0.73 (0.04-1.43)  1.26 (0.31-2.22)  2.54 (0.76-4.33)  0 [Reference]  0.40 (-0.31-1.11) |
| **Mixed Effects**^4^ |  | Any definite AD  No problem/mild  Moderate  Severe | 0.64 (0.43-0.85)  0.36 (0.14-0.58)  0.84 (0.51-1.17)  1.71 (1.05-2.36) | 0.31 (0.08-0.55)  0.17 (-0.07-0.42)  0.58 (0.21-0.95)  1.71 (0.94-2.47) |

Notes: ^1^ Predicted unit increase in SMFQ score (ranging from 0-26) among those with Atopic Dermatitis

^2^ Cross-sectional associations from adjusted multivariate linear regression models at each age. Models were adjusted for child sex, and ethnicity, maternal age at delivery, educational qualification (highest of either parent), social class based on occupation (highest of either parent), household crowding index, financial difficulties score, maternal pre- or post-natal depression, maternal pre- or post-natal anxiety and comorbid atopic diseases (asthma and allergic rhinitis) ^3^ No severity data available at age 18 years. ^4^ Adjusted multivariate mixed effects regression models using data from all ages listed in the table.

**eTable 5.** Longitudinal Associations with Symptoms of Depression (SMFQ) and Internalizing Behaviors (SDQ) by Missing Data Status

| **Mental health outcome**^1^  (questionnaire) | **AD activity and severity** | **Odds Ratio (95% CI)** | |
| --- | --- | --- | --- |
|  |  | **Adjusted for all covariates** | **Adjusted for covariates missing <1% of data^2^** |
| **Symptoms of Depression** (SMFQ) | Never  Any definite AD  No problem/mild  Moderate  Severe | 1 [Reference]  1.14 (0.93-1.40)  1.13 (0.88-1.46)  1.13 (0.78-1.65)  2.38 (1.21-4.72) | 1 [Reference]  1.13 (0.94-1.35)  1.04 (0.82-1.28)  1.22 (0.87-1.69)  1.99 (1.08-3.65) |
| **Internalizing Behavior** (SDQ) | Never  Any definite AD  No problem/mild  Moderate  Severe | 1 [Reference]  1.41 (1.17-1.70)  1.29 (1.06-1.57)  1.84 (1.40-2.41)  1.90 (1.14-3.16) | 1 [Reference]  1.47 (1.29-1.69)  1.41 (1.22-1.62)  1.66 (1.36-2.04)  1.73 (1.21-2.48) |

Notes: ^1^ Both the Short Moods and Feelings Questionnaire (SMFQ) and the Parent-reported Strength and Difficulties Questionnaire (SDQ) are modeled as a binary score. ^2^ Models were adjusted for covariates for which we have <1% missing data: child sex, age, maternal age at delivery, maternal pre- or post-natal depression, maternal pre- or post-natal anxiety and comorbid asthma.

**eTable 6.** Cross-sectional Associations with Clinician Diagnoses of Depression and Anxiety (based on the DAWBA)

| **Diagnosis**^1^ **(No.)** | **AD activity and severity** | **Odds Ratio (95% CI)** | |
| --- | --- | --- | --- |
|  |  | **Unadjusted** | **Adjusted** |
| Any Depressive Dx (41/7,338) | Never  Any definite AD  No problem/mild  Moderate  Severe | 1 [Reference]  6.10 (1.65-22.55)  3.66 (0.82-16.37)  9.88 (1.98-49.19)  13.22 (1.36-128.72) | 1 [Reference]  6.18 (1.27-30.17)  4.41 (0.78-24.76)  9.89 (1.54-63.41)  -----^2^ |
| Any Anxiety Dx (256/7,338) | Never  Any definite AD  No problem/mild  Moderate  Severe | 1 [Reference]  1.57 (1.07-2.29)  1.16 (0.73-1.83)  2.20 (1.22-3.98)  4.65 (2.05-10.54) | 1 [Reference]  1.53 (0.78-2.41)  1.01 (0.57-1.76)  2.53 (1.28-4.98)  5.60 (2.22-14.14) |
| Any Anxiety or Depressive Dx  (273/7,338) | Never  Any definite AD  No problem/mild  Moderate  Severe | 1 [Reference]  1.56 (1.08-2.26)  1.15 (0.73-1.79)  2.26 (1.27-4.01)  4.43 (1.96-10.04) | 1 [Reference]  1.51 (0.97-2.36)  1.00 (0.58-1.73)  2.53 (1.31-4.87)  5.18 (2.06-13.04) |

Notes: ^1^ Clinician-diagnosed outcomes based on the Development and Well Being Assessment (DAWBA), available at age 7 years.[2] This interview was comprised of open and closed questions about their child’s mental health that closely followed the diagnostic criteria in the DSM-IV or the ICD-10, and was reviewed by experienced clinicians who rated the presence or absence of ‘Any Depressive Disorder’ and ‘Any Anxiety Disorder’. ^2^ Insufficient data available for individuals with completed DAWBA and with ‘severe’ atopic dermatitis this age.

**eTable 7.** Associations Between Inflammatory Biomarkers and Symptoms of Depression (SMFQ) and Atopic Dermatitis

| **Biomarker^1^**  **(age)** | **Odds of Symptoms of Depression^2^**  **(OR, 95% CI)** | **Atopic Dermatitis**  **Adjusted Biomarker Coefficient^3^ (95% CI)** | |
| --- | --- | --- | --- |
| IL-6  (9 years) | 0.98 (0.85-1.13) | Never  Any definite AD  No problem/mild  Moderate  Severe | 0 [Reference]  0.12 (-0.01-0.25)  0.09 (-0.05-0.23)  0.23 (-0.02-0.47)  0.22 (-0.31-0.73) |
| CRP  (9 years) | 1.03 (0.92-1.14) | Never  Any definite AD  No problem/mild  Moderate  Severe | 0 [Reference]  0.02 (-0.15-0.19)  0 (-0.18-0.18)  0.15 (-0.17-0.47)  -0.20 (-0.88-0.48) |
| CRP  (16 years) | 0.93 (0.82-1.05) | Never  Any definite AD  No problem/mild  Moderate  Severe | 0 [Reference]  -0.03 (-0.27-0.22)  -0.05 (-0.34-0.24)  0.06 (-0.31-0.43)  -0.326(-1.01-0.49) |
| CRP  (18 years) | 0.93 (0.84-1.04) | Never  Any definite AD | 0 [Reference]  -0.17 (-0.46-0.13) |

Notes: ^1^ Biomarker values were log transformed. ^2^Cross-sectional associations from adjusted multivariate logistic regression models between biomarkers and symptoms of depression modeled as a binary outcome (a score of >11 on the Short Moods and Feelings Questionnaire). Models were adjusted for child sex, age, and ethnicity, maternal age at delivery, educational qualification (highest of either parent), social class based on occupation (highest of either parent), household crowding index, financial difficulties score, maternal pre- or post-natal depression, maternal pre- or post-natal anxiety and comorbid atopic diseases (asthma and allergic rhinitis) ^2^ Cross-sectional associations from adjusted multivariate linear regression models between atopic dermatitis and biomarker values. Coefficients represent the predicted unit change in the biomarker value with atopic dermatitis. Models were adjusted for child sex, age, and ethnicity, maternal age at delivery, educational qualification (highest of either parent), social class based on occupation (highest of either parent), household crowding index, financial difficulties score, maternal pre- or post-natal depression, maternal pre- or post-natal anxiety and comorbid atopic diseases (asthma and allergic rhinitis).**eTable 8.** Associations Between Sleep Quality and Internalizing Behaviors (SDQ) and Atopic Dermatitis

| **Sleep Quality^1^**  **(age)** | **Internalizing Behavior^2^**  **(aOR, 95% CI)** | **Atopic Dermatitis^3^**  **Adjusted Biomarker Coefficient (95% CI)** | |
| --- | --- | --- | --- |
| 4 years  7 years  9 years | 1.29 (1.21-1.38)  1.61 (1.50-1.73)  1.31 (1.22-1.41) | Never  Any definite AD  No problem/mild  Moderate  Severe  Never  Any definite AD  No problem/mild  Moderate  Severe  Never  Any definite AD  No problem/mild  Moderate  Severe | 0 [Reference]  0.17 (0.09-0.24)  0.14 (0.05-0.22)  0.18 (0.06-0.31)  0.38 (0.15-0.61)  0 [Reference]  0.09 (0.01-0.16)  0.03 (-0.05-0.12)  0.23 (0.08-0.37)  0.34 (0.05-0.62)  0 [Reference]  0.10 (0.02-0.19)  0.07 (-0.02-0.16)  0.19 (0.03-0.34)  0.34 (0.04-0.65) |

Notes: ^1^ Sleep quality was measured using 4 standardized questions asking about regular nighttime awakenings (≥1 per night), regular early morning awakenings, difficulty falling asleep, and nightmares over the past year. Responses were combined into a composite sleep-quality score ranging from 0 to 4. ^2^ Cross-sectional associations from adjusted multivariate logistic regression models. Odds of symptoms of internalizing behvaior defined as an emotional symptoms score of >4 on the Strength and Difficulties Questionnaire. ^3^ Cross-sectional associations from adjusted multivariate linear regression models. Coefficients represent the predicted unit increase in composite sleep-quality score (ranging from 0-4) among those with Atopic Dermatitis. All Models were adjusted for child sex, age, and ethnicity, maternal age at delivery, educational qualification (highest of either parent), social class based on occupation (highest of either parent), household crowding index, financial difficulties score, maternal pre- or post-natal depression, maternal pre- or post-natal anxiety and comorbid atopic diseases (asthma and allergic rhinitis).

**eTable 9.** Mediation by Sleep Quality of Associations Between Atopic Dermatitis and Internalizing Behaviors (SDQ)

| **Age, y** | **AD activity and severity** | **Odds Ratio (95% CI)** | | |
| --- | --- | --- | --- | --- |
|  |  | **Total effect (unadjusted for sleep quality)** | **Direct effect**  **(adjusted for sleep quality)** | **% Mediated**  **(95% CI)** |
| 4  7  9 | Never  Any definite AD  No problem/mild  Moderate  Severe  Never  Any definite AD  No problem/mild  Moderate  Severe  Never  Any definite AD  No problem/mild  Moderate  Severe | 1 [Reference]  1.49 (1.22-1.83)  1.41 (1.11-1.78)  1.59 (1.15-2.19)  1.99 (1.17-3.40)  1 [Reference]  1.42 (1.15-1.74)  1.29 (1.02-1.63)  1.81 (1.27-2.57)  1.90 (0.98-3.68)  1 [Reference]  1.25 (0.98-1.60)  1.24 (0.95-1.62)  1.07 (0.66-1.75)  2.69 (1.06-6.77) | 1 [Reference]  1.43 (1.16-1.75)  1.36 (1.08-1.73)  1.52 (1.10-2.10)  1.76 (1.02-3.02)  1 [Reference]  1.30 (1.04-1.62)  1.20 (0.94-1.53)  1.59 (1.10-2.30)  1.67 (0.84-3.34)  1 [Reference]  1.00 (0.80-1.26)  0.84 (0.65-1.10)  1.64 (1.13-2.39)  1.26 (0.60-2.64) | 11.32 (7.03 – 23.14)  10.48 (5.84 – 30.96)  10.67 (5.60 – 33.81)  16.65 (7.44 – 77.07)  14.67 (8.57- 40.21)  9.87 (4.02 – 51.81)  20.49 (11.95 – 49.71)  21.58 (-68.99 – 130.83)  28.52 (-563.63 – 371.40)  13.7 (-132.41 – 121.89)  16.23 (6.99 – 74.44)  17.92 (-154.28 – 196.45) |

**eFigure 1.** Study Participant Flow Diagram

**
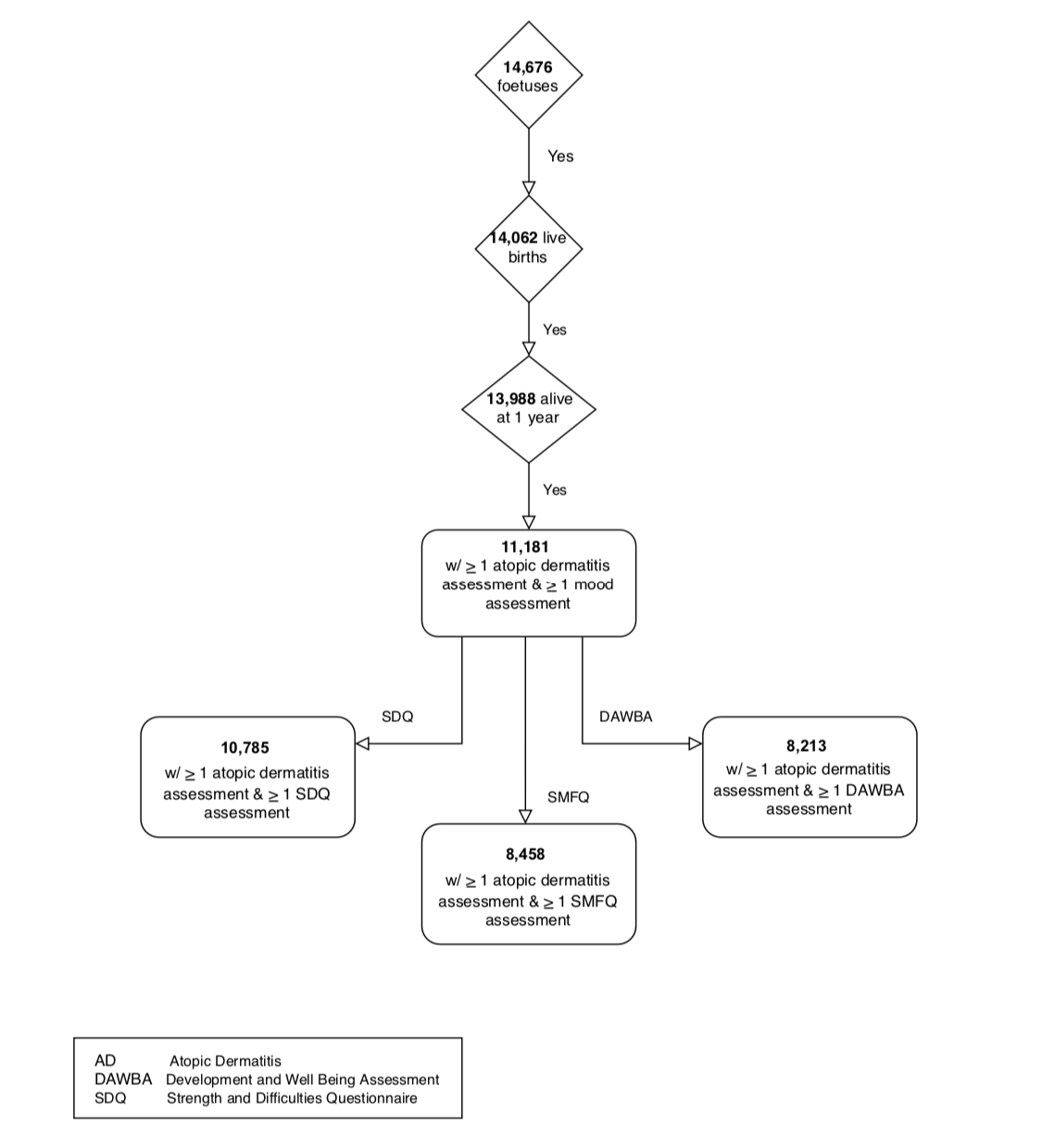
**

**eFigure 2.** Directed Acyclic Graph [3]

**
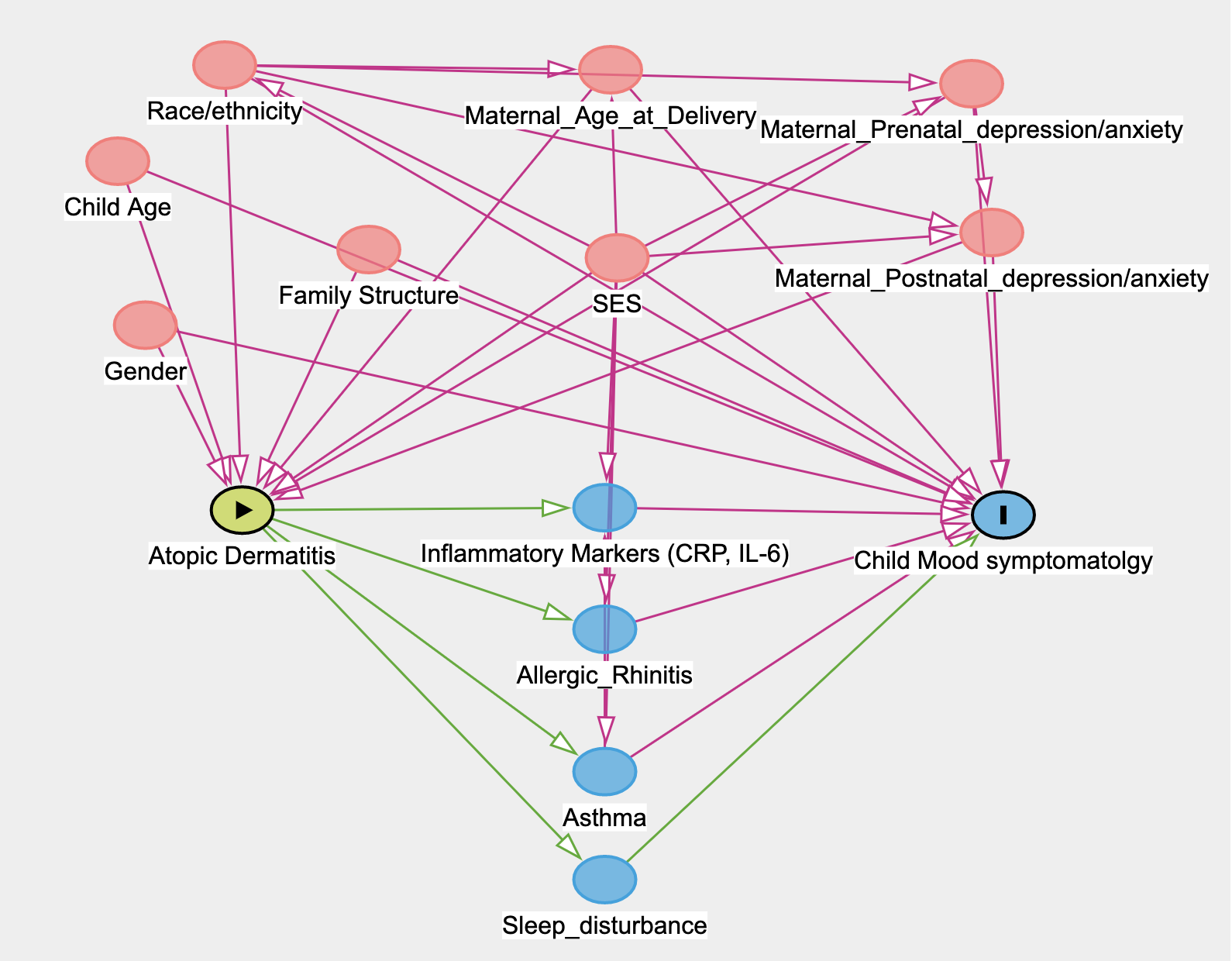
**

Notes: Potential confounding variables included: (1) sociodemographic characteristics: child sex, age, and ethnicity, and maternal age at delivery; (2) measures of socioeconomic status: educational qualification (highest of either parent), social class based on occupation (highest of either parent), household crowding index (number of people living in the household divided by the number of rooms in the house), and a financial difficulties score assessing the mother’s self-reported difficulty to afford food, clothing, heating, rent or mortgage, or other items necessary to care for the child; and (3) maternal mental health history: maternal pre- and post-natal depression defined as a score of 13 or greater on the Edinburgh Postnatal Depression Scale [4], and pre- and post-natal anxiety as a score of 8 or above on the Crown Crisp Anxiety scale at 18 weeks gestation and 8 months postpartum [5].

Potential mediating variables included: (1) comorbid atopy: defined as asthma or allergic rhinitis measured by the mother’s reports of asthma and/or allergic rhinitis symptoms at that time point, based on standardized questions similar to those used in the ISAAC study [6]; (2) sleep disturbance: assessed by sleep duration and sleep quality using standardized questionnaires at 5 time points (ages 4, 7, 9, 11, and 14 years) and 3 time points (ages 4, 7, and 9 years), respectively. Sleep duration was calculated by adding mother-reported nighttime and daytime sleep duration until age 7 and nighttime sleep duration from age 7 to 16. Sleep quality was measured using 4 standardized questions asking about regular nighttime awakenings (≥1 per night), regular early morning awakenings, difficulty falling asleep, and nightmares over the past year. Responses were combined into a composite sleep-quality score ranging from 0 to 4, assigning 1 point for each item as has been described elsewhere [7]; (3) inflammatory biomarkers: serum IL-6 (at age 9 only) and serum CRP (at ages 9, 14, and 16), both measured from serum samples collected during in-person clinic assessments. CPR and IL-6 serum samples from individuals with report of infection within 3 weeks of serum sample collection were excluded. CRP and IL-6 data were normalized by log-transformation for all analyses.

**e References**

1. Goodman, R., *Psychometric properties of the strengths and difficulties questionnaire.* J Am Acad Child Adolesc Psychiatry, 2001. 40(11): p. 1337-45.

2. Goodman, R., et al., *The Development and Well-Being Assessment: description and initial validation of an integrated assessment of child and adolescent psychopathology.* J Child Psychol Psychiatry, 2000. 41(5): p. 645-55.

3. Textor, J., et al., *Robust causal inference using directed acyclic graphs: the R package 'dagitty'.* Int J Epidemiol, 2016. 45(6): p. 1887-1894.

4. Pearson, R.M., et al., *Prevalence of Prenatal Depression Symptoms Among 2 Generations of Pregnant Mothers: The Avon Longitudinal Study of Parents and Children.* JAMA Netw Open, 2018. 1(3): p. e180725.

5. Capron, L.E., et al., *Associations of maternal and paternal antenatal mood with offspring anxiety disorder at age 18 years.* J Affect Disord, 2015. 187: p. 20-6.

6. Asher, M.I., et al., *International Study of Asthma and Allergies in Childhood (ISAAC): rationale and methods.* Eur Respir J, 1995. 8(3): p. 483-91.

7. Ramirez, F.D., et al., *Association of Atopic Dermatitis With Sleep Quality in Children.* JAMA Pediatr, 2019. 173(5): p. e190025.
